# A Personalized, Risk-Based Approach to Active Surveillance for Prostate Cancer with Takeaways from Broader Oncology Practices: A Mixed Methods Review

**DOI:** 10.3390/jpm15030084

**Published:** 2025-02-25

**Authors:** Jeroen J. Lodder, Sebastiaan Remmers, Roderick C. N. van den Bergh, Arnoud W. Postema, Pim J. van Leeuwen, Monique J. Roobol

**Affiliations:** 1 Department of Urology, Erasmus MC Cancer Institute, University Medical Center Rotterdam, 3015 GD Rotterdam, The Netherlands; s.remmers@erasmusmc.nl (S.R.); r.vandenbergh@erasmusmc.nl (R.C.N.v.d.B.); m.roobol@erasmusmc.nl (M.J.R.); 2 Department of Urology, Leiden University Medical Center, 2333 ZA Leiden, The Netherlands; a.w.postema@lumc.nl; 3 Department of Urology, Netherlands Cancer Institute, Antoni van Leeuwenhoek Hospital, 1066 CX Amsterdam, The Netherlands

**Keywords:** active surveillance, prostatic neoplasms, risk prediction

## Abstract

**Background/Objectives**: To summarize the current state of knowledge regarding personalized, risk-based approaches in active surveillance (AS) for prostate cancer (PCa) and to explore the lessons learned from AS practices in other types of cancer. **Methods**: This mixed methods review combined a systematic review and a narrative review. The systematic review was conducted according to the Preferred Reporting Items for Systematic rviews and Meta-Analyses (PRISMA) guidelines, with searches performed in the Medline, Embase, Web of Science, Cochrane Central Register of Controlled Trials, and Google Scholar databases. Only studies evaluating personalized, risk-based AS programs for PCa were included. The narrative review focused on AS approaches in other solid tumors (thyroid, breast, kidney, and bladder cancer) to contextualize the findings and highlight lessons learned. **Results**: After screening 3137 articles, 9 were suitable for inclusion, describing the following four unique risk-based AS tools: PRIAS, Johns Hopkins, Canary PASS, and STRATCANS. These models were developed using data from men with low-risk (Grade Group 1) disease, with little to no magnetic resonance imaging (MRI) data. They used patient information such as (repeated) prostate-specific antigen (PSA) measurements and biopsy results to predict the risk of upgrading at the next biopsy or at radical prostatectomy, or to assign a patient to a pre-defined risk category with a corresponding pre-defined follow-up (FU) regimen. Performance was moderate across models, with the area under the curve/concordance index values ranging from 0.58 to 0.85 and calibration was generally good. The PRIAS, Canary PASS, and STRATCANS models demonstrated the benefits of less burdensome biopsies, clinic visits, and MRIs during FU when used, compared to current one-size-fits-all practices. Although little is known about risk-based AS in thyroid, breast, kidney, and bladder cancer, learning from their current practices could further refine patient selection, streamline monitoring protocols, and address adoption barriers, improving AS’s overall effectiveness in PCa management. **Conclusions**: Personalized, risk-based AS models allow for a reduction in the FU burden for men at low risk of progression while maintaining sensitive FU visits for those at higher risk. The comparatively limited evidence and practice of risk-based AS in other cancer types highlight the advanced state of AS in PCa.

## 1. Introduction

Prostate cancer (PCa) is the second most frequently diagnosed cancer among men worldwide [1], representing a significant public health concern. Despite advances in PCa diagnostic algorithms, including the adoption of risk-adapted approaches using risk calculators [2] and Magnetic Resonance Imaging (MRI) [3], 36% of newly diagnosed tumors are still considered clinically insignificant (overdiagnosed) [4], meaning they would not have caused any clinical consequences during a man’s lifetime if left untreated. To mitigate the overtreatment of these clinically insignificant tumors, patients are often recommended to participate in active surveillance (AS) programs [5,6]. AS involves a combination of prostate-specific antigen (PSA) testing, digital rectal examinations (DRE), MRI, and prostate biopsies to closely monitor a patient’s disease status. AS is based on the premise that curative treatment can still be administered within a critical timeframe if necessary. This strategy allows for the postponement or even avoidance of definitive treatment and its associated side effects, thereby reducing overtreatment [7].

The safety of AS for low-risk PCa is well established, with a cancer-specific mortality rate of 1.5% and a metastatic disease rate of 2.8% after 15 years of follow-up (FU) [8]. More recently, AS for well-selected patients with intermediate-risk PCa has gained acceptance [7]. However, these ‘high-risk’ surveillance candidates might need more intensive monitoring than the low-risk surveillance candidates. This is highlighted by the recent 15-year update of the ProtecT trial [9], which showed that while 9.4% of men in the active monitoring group developed metastatic disease compared to 4.8% in the active treatment group, there was no overall survival benefit for active treatment. Importantly, 34% of the cohort were intermediate- or high-risk patients. Furthermore, the monitoring protocol in this study relied solely on PSA testing without protocolized imaging or repeated biopsies. These long-term results in a mixed cohort, all monitored with relatively low-intensity FU, raise questions about which patients would benefit from the current, more intensive AS protocols, and which patients could safely be monitored less intensively.

Currently, there is no consensus on the optimal frequency of surveillance assessments in AS or on when an AS program is considered safe enough. Most contemporary AS protocols adopt a one-size-fits-all approach with regular, frequent PSA measurements, MRIs, and repeat biopsies. While biopsies remain the gold standard for detecting disease progression or reclassification, they are burdensome and carry risks of infection and bleeding [10]. Additionally, the anticipation of repeat tests and the possibility of discovering cancer progression can increase patient anxiety and therefore impact quality of life [11,12]. This combination of physical and emotional strain can reduce compliance with AS protocols, potentially leading to the delayed detection of disease progression and worse oncologic outcomes [13,14,15].

Given the heterogeneous group of patients admitted to AS [16], a uniform monitoring schedule is not clinically justified, nor aligned with the principles of patient-centered care. A promising alternative that takes this into account is personalized, risk-based monitoring, which uses individual clinical data to tailor surveillance schedules according to the patient’s risk of disease progression. This approach seeks to minimize unnecessary FU measurements while ensuring the timely detection of progression for those who need it. The recent expert consensus highlights the development of such an approach with less frequent testing for men at the lowest risk of progression as a top priority for research [17].

AS has also been applied in other types of cancer. For instance, AS is now a recognized option in multiple guidelines for thyroid [18] and kidney cancer [19], and it is still being explored in stage 0 breast cancer (ductal carcinoma in situ) [20] and bladder cancer [21]. These practices underscore the importance of monitoring low-risk cancers, balancing the avoidance of unnecessary interventions with timely treatment for those who need it [22]. To summarize the current state of knowledge regarding personalized, risk-based approaches in AS for PCa and to explore lessons learned from AS practices in other types of cancer, we conducted this mixed methods review. By evaluating the performance and limitations of risk-based surveillance tools we hope to enhance our understanding of their potential role in optimizing AS protocols and minimizing unnecessary and sometimes burdensome FU measurements.

## 2. Materials and Methods

This mixed methods review combined a systematic review and a narrative review. The reporting of this systematic review was guided by the standards of the Preferred Reporting Items for Systematic Reviews and Meta-Analyses (PRISMA) statement. We defined personalized, risk-based monitoring programs as tools that utilize patients’ repeated measurements and characteristics to predict the risk of a specific event of interest (e.g., disease upgrading, discontinuation of AS, or adverse pathology following radical prostatectomy (RP)) or to determine the next step in FU (e.g., algorithms). The narrative review focused on AS approaches in other solid tumors (thyroid, breast, kidney, and bladder cancer) to contextualize the findings and highlight lessons learned.

### 2.1. Search Strategy

The search terms and comprehensive literature search strategy were developed and executed in collaboration with an information specialist (CN). We queried the Medline, Embase, Web of Science Core Collection, Cochrane Central Register of Controlled Trials, and Google Scholar databases in May 2024, to identify studies addressing personalized, risk-based active surveillance. The search strategy incorporated a combination of medical subject headings (MeSH terms) and keywords related to “prostate cancer”, “active surveillance”, and “risk model”. Language restrictions were applied, limiting the review to articles in English. Duplicate studies were removed before screening. Articles were imported into the Covidence workflow management platform [23]. The complete search strategy can be found in Table A1 in Appendix A.

### 2.2. Study Selection

Two reviewers (JL and SR) performed an independent initial screening based on titles and abstracts. Full texts of potentially relevant studies were retrieved and evaluated for eligibility based on predefined inclusion and exclusion criteria. Discrepancies were resolved by consensus among the reviewers. Reviews, comments, replies, and study protocols were excluded.

### 2.3. Inclusion and Exclusion Criteria

#### 2.3.1. Population

Participants in the included studies were required to be adult men (>18 years of age) with a diagnosis of low- or intermediate-risk PCa monitored with AS. Studies regarding PCa screening and studies involving patients without a diagnosis of PCa, patients with metastatic PCa, patients with biochemical recurrence of PCa, and patients on watchful waiting were excluded.

#### 2.3.2. Intervention and Comparison

The inclusion criteria encompassed studies specifically dedicated to the development and validation of personalized predictive models and algorithms in the context of AS. Studies about risk calculators in the context of PCa detection were excluded.

#### 2.3.3. Outcomes

The outcomes of interest centered on the performance of personalized, risk-based tools to predict a specific event of interest or to define the next step in the FU within the context of AS. There were no restrictions regarding the AS protocols or predictors used. The review excluded studies that exclusively examined risk factors for certain outcomes of AS (e.g., disease upgrading, discontinuation of AS, and adverse pathology following RP), studies that utilized a single measurement to predict a certain event of interest, and genetic risk models.

#### 2.3.4. Data Extraction

A data extraction form was used to retrieve data from the included studies. The extracted data encompassed various elements, including study characteristics such as author, publication year, and study design. The developed or validated models described in the studies were documented together with their included variables, type of statistical model, and the development population. Outcome measures were recorded, including key metrics such as the area under the curve (AUC), to provide a quantifiable assessment of the model discrimination. Additionally, calibration evaluation metrics, including the calibration slope, calibration intercept, and graphical calibration plots, were extracted to offer insights into the alignment between predicted probabilities and observed outcomes. When available, the estimated difference in burden and benefit between personalized biopsy schedules following the prediction model and fixed biopsy schedules were reported.

#### 2.3.5. Data Synthesis and Analysis

The extracted data were collected per study to provide an overview of the development and validation procedures. We examined the performance of the models using the AUC, calibration evaluation, and/or the estimated burden and benefit of different biopsy schedules, depending on the available data.

## 3. Results

**Figure 1:**
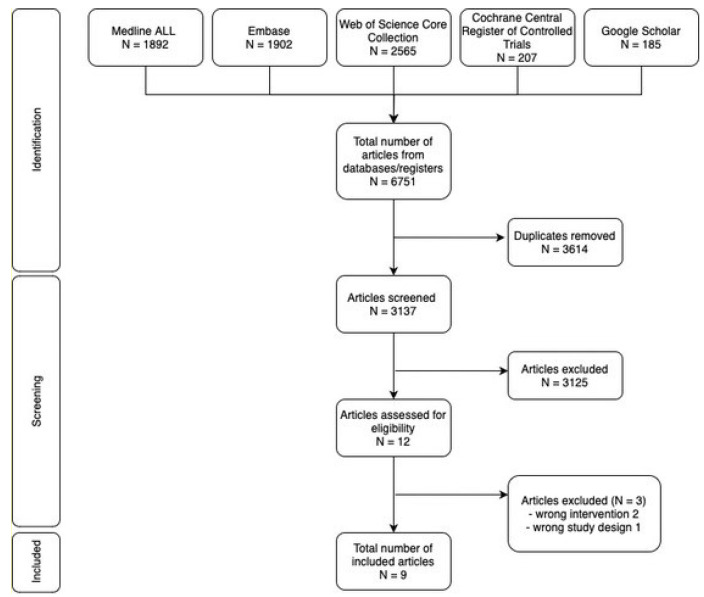
PRISMA flowchart of the review search.

The literature search comprised 6751 articles. After removing duplicates, 3137 articles remained, and 9 were included after screening the titles/abstracts and full texts [24,25,26,27,28,29,30,31,32]. Figure 1 illustrates the PRISMA flowchart for the study selection. We identified four unique risk-based AS tools from the nine included studies as follows: Prostate Cancer Research International Active Surveillance (PRIAS), Johns Hopkins, Canary Prostate Active Surveillance Study (Canary PASS) and STRATified CANcer Surveillance (STRATCANS). Below, these four models will be described separately.

### 3.1. PRIAS Model

The PRIAS model, first described in 2019 [30], was developed using data from 5270 patients in the PRIAS study. All patients had GG1 disease, did not undergo MRI, and were diagnosed with only systematic biopsies. This model used patients’ longitudinal PSA and biopsy measurements, as well as their Gleason Score at diagnosis, to predict the risk of cancer progression to GG2 or higher upon repeat biopsy through a bivariate joint modeling approach. Based on these risk predictions, the model offered a personalized, risk-adapted biopsy schedule, tailored to each patient.

After internal validation, the model underwent external validation in the largest six AS cohorts from the Movember Foundation’s third Global Action Plan (GAP3) database [29]. It demonstrated moderate performance in all cohorts, with AUC values ranging from 0.58 to 0.79. Model calibration was shown using calibration plots. The model showed signs of miscalibration, likely due to differences in the inclusion criteria and FU protocols, requiring the recalibration of the baseline hazard. After recalibration, the predicted outcomes aligned well with the observed data, particularly in cohorts where PSA’s impact on the upgrading risk was similar to that in PRIAS. Model validation relied on retrospective analyses, which may have introduced bias related to patient selection and data completeness. Ultimately, the model’s personalized schedules suggested an average of six fewer biopsy procedures over 10 years compared to an annual schedule, and two fewer biopsies than the PRIAS standard schedule, while maintaining almost the same time delay in detecting upgrading (Table 1).

### 3.2. Johns Hopkins Model

The Johns Hopkins model, first described in 2017 [25], was developed using data from 964 patients from the Johns Hopkins Active Surveillance (JHAS) cohort who met the Epstein criteria for very low-risk PCa and had at least two PSA measurements and one postdiagnosis biopsy as of 1 January 2016 [33]. Patients did not undergo MRI at diagnosis. This model used patients’ longitudinal PSA and biopsy measurements to predict the Pathologic Gleason Score (PGS) after RP in four ordered categories as follows: 6 (GG 1), 3 + 4 (GG 2), 4 + 3 (GG 3), and 8–10 (GG 4 and 5). A Bayesian joint modeling approach was used to achieve these predictions. Internal validation in the JHAS cohort demonstrated moderate performance, with AUC values ranging from 0.63 to 0.75. Calibration plots indicated that PGS predictions were closely aligned with the observed post-surgery PGS rates. There were no data on the potential reduction in FU visits or biopsies if this model were to be implemented. The model was not externally validated, limiting its generalizability to broader populations.

### 3.3. Canary PASS Model

The Canary model, first described in 2020 [27], was developed using data from 850 men with GG1 disease, enrolled in the Canary PASS cohort—a multicenter prospective cohort study of men on AS at nine North American centers. Most men did not undergo MRI. This model used patients’ longitudinal PSA and biopsy measurements, time since diagnosis, BMI, and prostate size to predict the risk of reclassification, defined as any increase to GG2 or higher. It did so by assessing the time from a biopsy or PSA measurement until reclassification, using a time varying Cox regression.

The model was externally validated in a separate cohort of 553 men with GG1 disease from the University of California, San Francisco (UCSF) who were not part of the PASS cohort. For predicting non-reclassification at 4 years after confirmatory biopsy, the model demonstrated moderate performance, with an AUC of 0.70 in both the PASS and UCSF cohorts. Calibration plots indicated good calibration for both cohorts. Theretrospective nature of the validation, together with the lack of validation in patients not living in the San Francisco region, may have introduced patient selection bias and data incompleteness, resulting in a lack of generalizability. When applied, the model suggested that among men in the lowest decile of risk (with a risk threshold of 0.08 at the 10th percentile), omitting all surveillance for 4 years would miss only five reclassification events per 1000 men (95% confidence interval (CI): 0–11). In the lowest quartile of risk (risk threshold of 0.13 at the 25th percentile), this number would increase to 29 missed events per 1000 men (95% CI: 16–42) (Table 2).

### 3.4. Stratcans Model

The STRATCANS model, first described in 2019 [34], combines the Cambridge Prognostic Groups (CPG), which are based on diagnostic data alone (clinical stage, Gleason Score, and PSA level [35]), with PSA-density (PSAD). It stratifies patients into three risk-groups based on their risk of progression to CPG 3 disease (i.e., localized disease with GG2 and a PSA between 10 and 20 ng/mL, or localized disease with GG3 and a PSA < 20 ng/mL), each with their own FU intensity (Table 3). The model allows for re-stratification over time as new FU data become available.

The model underwent internal and external validation [36]. Discrimination was good in both the development (concordance index (C-index) 0.749), internal validation (C-index 0.742) and external validation (C-index 0.845), and the calibration plots indicated good calibration, with calibration slopes ranging from 0.982 to 0.944. Although multicenter, the development cohort was modestly sized (n = 883), and the external cohort was small (n = 151). There were differences between cohorts in AS eligibility criteria, practice, and available FU time, introducing heterogeneity, which may have affected the model’s performance. In a study reporting early outcomes from STRATCANS implementation [28], its protocol was compared to the NICE guideline recommendations for AS, which involve annual clinical reviews with DRE, repeat MRI at 12 months, and similar PSA monitoring intervals. For a cohort of 126 men with 12 months of FU, adherence to the NICE schedule would require a total of 126 clinic visits and 126 MRI scans, compared to 98 clinic visits (−22%) and 73 MRI scans (−42%) when following the STRATCANS protocol (Table 4).

### 3.5. Head-to-Head Comparison

A direct, head-to-head comparison of the PRIAS, Johns Hopkins, Canary, and STRATCANS models was not feasible, as each model was designed to predict different clinical outcomes and used distinct measures of potential benefit. Additionally, reductions in clinical burden were measured differently across models, with benefits defined as decreases in clinic visits, MRIs, or biopsies, measured over varying FU periods. Nevertheless, Table 5 shows a concise overview of all four models for reference.

## 4. Insights from Other Cancer Types

### 4.1. Thyroid Cancer

Papillary thyroid microcarcinoma (PTMC), defined as a tumor ≤ 1 cm in size, has an excellent prognosis, with disease-specific mortality below 0.1% and a recurrence rate of approximately 3%. When monitored without immediate surgery, most PTMCs (>80%) remain stable, with no change in size. This indolent nature has led to the emergence of AS as a management strategy, helping prevent overtreatment and its associated side effects, such as thyroid hormone imbalance or voice changes due to surgical complications [37].

The management of PTMC using AS is increasingly guided by a risk-stratified framework, which assesses the following three domains: ultrasound characteristics (e.g., tumor size, location, molecular profile, and nodal involvement), patient factors (e.g., age, family history, willingness to defer surgery, and compliance with FU), and medical team expertise (e.g., access to high-quality imaging and experienced clinicians). Patients are categorized as ideal, appropriate, or inappropriate candidates for AS. Ideal candidates, such as older patients with solitary, well-defined tumors, have a low risk of progression (<1–2%) and benefit from therapy in case of disease progression. Appropriate candidates, with higher-risk features, face a moderate risk of progression (~10%), but can still achieve excellent outcomes with careful monitoring. Inappropriate candidates, including those with critical tumor locations or metastasis, require immediate treatment [38].

Despite advancements in candidate selection, all PTMC patients under AS typically follow uniform monitoring protocols, often involving regular ultrasound imaging (every 6–12 months) [39,40,41]. To date, there is no literature on a personalized, risk-based approach to adjust the intensity of monitoring over time based on individual patient risk.

### 4.2. Breast Cancer

Ductal carcinoma in situ (DCIS) is a potential precursor to invasive breast cancer (IBC). Its incidence has increased with the introduction of population-based breast screening and digital mammography, with approximately 25% of all screen-detected breast cancers now being DCIS. As DCIS has the potential to progress to IBC, it is typically treated like early-stage IBC. Current treatment guidelines recommend surgery, either mastectomy or breast-conserving surgery, often followed by radiotherapy and, in some countries, endocrine treatment. However, up to 80% of DCIS lesions are indolent, low-risk lesions that will not progress to IBC during the patient’s lifetime [42]. The long-term risk of dying of breast cancer in women diagnosed with DCIS is very low, at approximately 3% [43].

AS is emerging as a promising management option for DCIS, but the reliable identification of low-risk lesions and the prospective demonstration of its safety are essential for its widespread adoption. Four trials have been initiated, three of which are ongoing [44,45,46], and these are expected to provide critical evidence supporting the de-escalation of surgical treatment for selected patients with low-risk DCIS [43]. In these trials, AS involves of a mammography every 6–12 months, with or without a clinical breast examination. We could not identify any personalized, risk-based AS-programs for DCIS.

Interestingly, the LORD-trial [44] used a patient-preference design, allowing women to choose between AS or conventional treatment. This approach offered valuable insights into the preferences of women with low-risk DCIS, revealing a strong preference for AS (76%) compared to conventional treatment (24%) [42]. Women who chose AS reported their belief that immediate treatment was not (yet) necessary and expressed high levels of trust in the safety of AS. Conversely, those who chose conventional treatment seemed to be driven by a wish to avoid cancer worry. Notably, women opting for AS more often experienced shared decision making (SDM) compared to women opting for conventional treatment. These findings highlight the importance of comprehensive patient education and SDM in fostering confidence in AS; lessons that also resonate strongly with the management of PCa.

### 4.3. Kidney Cancer

There has been increased interest in AS as a management strategy for small renal masses (SRM), given evidence supporting their natural history is indolent, slow growing, and of limited metastatic potential. Despite the prominence of AS in the management of SRM, the practice of AS appears to rest on a handful of general principles, with plenty of room for interpretation left to the clinicians and with a strong emphasis on patient preference. More nuanced and evidence-based strategies for patient selection and surveillance have not been employed, much less personalized, risk-based strategies [47]. One of the research priorities identified as a part of a renal cancer-modified Delphi consensus statement for low-risk kidney cancer was developing an evidence-based active surveillance protocol based on the natural history of the SRM and the impact of different imaging protocols [48].

Currently, AS in kidney cancer involves monitoring tumor size by serial abdominal imaging (ultrasound, computed tomography, or MRI) with delayed intervention for tumors showing clinical progression during FU. However, the timing and frequency of imaging remain unspecified [49]. Unlike in thyroid and breast cancer, AS in kidney cancer particularly benefits elderly and comorbid patients, who often face low cancer-specific mortality but significant competing-cause mortality [50]. This underscores the importance of considering frailty as a critical risk factor in clinical decision making, given its impact on both perioperative and oncological outcomes. This aligns with the general belief that frail people with low-grade PCa benefit more from AS, or even watchful waiting, than from active treatment.

### 4.4. Bladder Cancer

AS is a developing management strategy for low-grade non-muscle invasive bladder cancer (NMBIC) to reduce overtreatment and associated morbidity. Although the majority of patients affected by NMBIC experience recurrence during FU, relatively few of them will progress to MIBC. Following this observation, Soloway et al. proposed AS is a safe and valid alternative to active treatment in 2003; and Miyaki et al. subsequently proposed a FU regimen for AS, using size and multifocality as triggers for intervention. However, the level of evidence in favor of AS is low, with observational studies having heterogeneous selection criteria, triggers for intervention, and surveillance tools [21,51,52]. Current guidelines, therefore, only recommend AS to carefully selected patients with a weak strength rating and without a clear FU regimen [21].

The multicenter prospective Bladder Cancer Italian Active Surveillance (BIAS) project is a pioneering prospective study investigating the feasibility, safety, and oncologic outcomes of AS in low-grade NMIBC. It enrolls patients who meet specific inclusion criteria, including small tumor size, low number of lesions, and no history of high-grade disease or muscle invasion, and closely monitors them with regular cystoscopic follow-ups, without the immediate surgical resection of recurrent lesions, unless specific progression criteria are met. Long-term results showed that after a median FU of 38.8 months [IQR 28.6–55.5], out of 251 AS events, 130 AS failures (51.8%) were observed, of which 9.2% and 0.7% showed a high-grade Ta/T1 and a T2 tumor, respectively. Furthermore, the treatment-free probability at 36 months was 40.4%. These results confirmed that well-selected patients with NMBIC can safely remain on AS for a long period of time [53].

Despite these promising results and the growing interest in AS as a management strategy for low-grade NMBIC, further large-scale, long-term studies are needed to establish standardized protocols and broaden the acceptance of AS in bladder cancer management. We could not identify any personalized, risk-based AS-programs for bladder cancer.

## 5. Discussion

AS has established itself as a cornerstone of management for low-risk PCa [54,55], with decades of evidence supporting its safety [9,56,57,58,59] and incorporation into international guidelines [5,6]. In contrast, AS is still emerging as a potential management strategy for other cancers, including thyroid, breast, kidney, and bladder cancers. This mixed methods review highlights the advanced state of AS in PCa, with recent innovations focused on personalized, risk-based approaches. These AS tools use patient information such as (repeated) PSA measurements and biopsy results to predict the risk of upgrading at the next biopsy or at RP, or to assign a patient to a pre-defined risk category with a corresponding pre-defined FU regimen. In comparison, the adoption of AS in other cancer types is less mature, marked by variable guideline recommendations and the absence of standardized FU protocols (thyroid and kidney cancer) or ongoing trials still aiming to establish its safety (breast and bladder cancer). Despite these differences, parallels exist, such as the critical role of patient selection and SDM.

An important limitation of most current AS protocol for PCa is their reliance on a one-size-fits-all approach with repeated PSA measurements, MRI, and prostate biopsies at fixed and predetermined intervals, irrespective of the individual’s risk. While these measures aim to ensure the timely detection of disease progression, they also impose significant burdens on patients, particularly repeat biopsies, which can lead to poor compliance and, consequently, worse oncologic outcomes [13,15]. Emerging evidence from PCa screening trials, such as the ProScreen trial [60] and the GOTEBORG-2 trial [61], further supports this shift toward more selective use of biopsies and the potential to safely delay or omit biopsies in certain low-risk scenarios. While these trials were conducted in a screening population rather than AS, their findings may inform future risk-based AS protocols.

Reducing unnecessary out-patient visits, MRI scans, and repeat biopsies is not only in the patient’s best interest, but it is also absolutely required to manage the increasing burden PCa is expected to exert on healthcare systems. The growing use of prostate MRI, together with increasing demands for urology outpatient visits and biopsies, has been shown to strain healthcare budgets and resources, leading to prolonged waiting times in some regions [62,63,64,65]. This, in combination with healthcare workforce shortages [66], highlights the pressing need for personalized, risk-based AS programs capable of de-intensifying FU regimens, both for PCa and for other cancer types.

In our review, we identified four unique risk-based AS tools that address these limitations, three of which underwent external validation, while one was only internally validated. All four models were developed using data from patients with low-risk PCa (GG1) disease without MRI data, and three of them demonstrated the potential to reduce patient burden without significant delays in detecting upgrading or missed cases of upgrading. The PRIAS model showed an average reduction in six biopsies measured over 10 years, compared to an annual biopsy schedule, and two fewer biopsies, compared to the standard PRIAS protocol in slow/non-progressing AS patients, while maintaining almost the same time delay in detecting upgrading [29,30,31,32]. The Canary PASS model demonstrated a reduction of 100 FU visits per 1000 lowest-risk men on AS, with only 5 (95% CI: 0–11) missed reclassifications [26,27], and the STRATCANS showed a 42% reduction in MRI usage and a 22% reduction in clinic visits [28]. Model performance varied but was generally moderate, with AUC values ranging between 0.58 and 0.79, C-index values ranging between 0.74 and 0.85, and showing good calibration. For comparison, a recent systematic review on risk calculators for the detection of PCa showed an average AUC of 0.75 for the Rotterdam Prostate Cancer Risk Calculator (RPCRC), one of the most used and most validated clinical tools in PCa management [2], and, in general, an AUC of >0.70 is considered acceptable [67]. Although these models collectively highlight the potential benefits of personalized, risk-based AS programs, the reductions in clinical burden were measured differently across the models, making a direct head-to-head comparison impossible. This variability underscores the need for consensus on standardized outcome measures, enabling cross-model comparisons in future studies and facilitating evidence-based decisions on the optimal approach to risk-based AS.

The biggest strength of the PRIAS, Johns Hopkins, and Canary PASS models is arguably their dynamic character, as they incorporate repeated measurements to continuously update risk predictions over time, using all the available data rather than solely relying on the most recent input. This dynamic approach potentially leads to more accurate risk predictions and has been endorsed as the preferred method of risk estimation by the 2023 consensus panel [17]. However, the applicability of these models is limited as they were developed using the data of low-risk patients with GG1 disease, with little to no MRI data. All models acknowledge this limitation and explain that it is due to the scarcity of long-term data from intermediate-risk (GG2) patients who underwent an MRI, while emphasizing the potential to incorporate these data into future models. Furthermore, the events of interest were upgrading at repeat biopsy or RP to GG2 or higher, which does not reflect contemporary AS practices, as GG2 has been accepted within many AS programs and upgrading to GG2 alone is no longer a reason to switch to active treatment [68]. While all three models predict the risk of upgrading, they do not provide clear thresholds for when an MRI or repeat biopsy should be performed, leaving the decision to clinical judgment. This flexibility could help in the personalization of every single choice in the AS program and stimulate SDM, but the absence of clear guidelines and thresholds could complicate the implementation of these models in clinical practice. Furthermore, for adequate implementation, these tools would need to be fully integrated into electronic health record systems to allow automatic updates with new patient data.

The STRATCANS model stands out for its pragmatic design, with a clear division into predefined risk-groups and FU intensities, making it highly standardized and implementable. This structured approach also simplifies research comparing different risk groups and FU intensities. However, like the dynamic models, STRATCANS was developed without incorporating MRI data, limiting its relevance to current AS practices. Additionally, this strategy is less flexible and offers less precision compared to dynamic risk calculators.

The limitations of these existing models raise the following critical questions: What outcomes should AS models predict? How many FU visits and measurements can we allow the healthcare system to do to miss one case of upgrading? And, ultimately, how much ‘worse’ are we willing to allow AS outcomes to be. compared to radical treatment. in exchange for reduced clinical burden and potentially better quality of life? These considerations are important when designing AS protocols that are both effective, patient-centered and sustainable for our healthcare system.

A critical area for future innovation in PCa management is the integration of emerging technologies such as molecular tests, MRI-based tools, artificial intelligence (AI), and liquid biopsies (LB) into AS protocols. Current risk-based models primarily rely on PSA levels, the Gleason Score, prostate volume, and clinical stage, yet they underutilize biopsy material. Molecular tests such as Decipher, Prolaris, and Oncotype DX have shown promise in improving prognostic accuracy, particularly for intermediate-risk PCa [69,70]. Similarly, MRI-based tools such as The Prostate Cancer Radiological Estimation of Change in Sequential Evaluation (PRECISE) score can refine AS monitoring strategies by estimating the likelihood of significant progression [71,72]. AI-driven tools could identify subtle changes in MRI images that may be overlooked by radiologists, offering more consistent interpretation and reducing interobserver variability [73]. LB could provide non-invasive options for monitoring tumor biology and help predict disease progression [74]. Incorporating these technologies into AS protocols could improve patient outcomes while minimizing the burden of FU procedures.

While current risk-based AS models already represent a significant step forward, their limitations underscore the need for new tools that incorporate data from intermediate-risk patients (GG2), serial MRI measurements, and emerging new technologies. By leveraging biopsy material, imaging data, and molecular insights, we can move towards a more comprehensive and personalized approach to AS. This next generation of AS protocols should be guided by a clear consensus of acceptable outcomes and the ‘price’ we are willing to pay for de-intensification, ensuring optimal care for patients while also alleviating the burden on healthcare systems and ensuring future PCa management remains possible.

## 6. Conclusions

Personalized, risk-based AS represents a promising strategy for optimizing FU intensity, reducing unnecessary outpatient visits, imaging, and repeat biopsies while maintaining favorable outcomes. However, current tools are limited by their reliance on data from low-risk populations and outdated definitions of upgrading. To fully realize the potential of risk-based AS, future models must integrate data from contemporary AS populations, as well as incorporate advancements in biomarkers and molecular testing. Continued research and consensus on standardized outcome measures are essential in refining these tools, ensuring they are both clinically relevant and sustainable within modern healthcare systems.

## Figures and Tables

**Table 1:** Burden and benefit PRIAS model measured over 10 years on AS.

Biopsy Scheme	Biopsy Sessions (No.)	Delay in Detection of Upgrading (Years)
Annual	10	0.5
PRIAS protocol	4	0.75
PRIAS model		
Risk 5%	6	0.6
Risk 10%	4	0.75
Risk F1	2	0.9

**Table 2:** Burden and benefit Canary PASS model measured over 4 years on AS.

Risk Threshold (Percentile)	Follow-Up Visits Avoided per 1000 Men	Reclassification Cases Missed per 1000 Men (95% CI)
0.08 (10th)	100	5 (0–11)
0.13 (25th)	250	29 (16–42)

**Table 3:** STRATCANS active surveillance risk-stratification system with follow-up [34].

Surveillance Group	Inclusion Criteria	Suggested Follow-Up
Low intensity	CPG 1 ^1^ PSAD < 0.15 ng/mL/mL Life expectancy 10 years	3–6 monthly PSA; 18 monthly out-patients MRI at 3 years (no lesion); MRI at 18 months (lesion seen) No routine biopsy; triggered re-biopsy if any change
Moderate intensity	CPG 2 ^2^ OR PSAD ≥ 0.15 ng/mL/mL Life expectancy 10 years	3–6 monthly PSA; 12 monthly out-patients MRI at 18 months (no lesion); MRI at 12 months (lesion seen) Re-biopsy at 3 years; triggered re-biopsy if any change
High intensity	CPG 2 ^2^ AND PSAD ≥ 0.15 ng/mL/mL Life expectancy 10 years	3–6 monthly PSA; 6 monthly out-patients MRI at 12 months Re-biopsy at 2 years; triggered re-biopsy if any change

^1^ CPG 1: GG1, PSA < 10 ng/mL, and clinical stage T1-T2. ^2^ CPG 2: GG2 or PSA 10–20 ng/mL and clinical stage T1-T2.

**Table 4:** Burden and benefit STRATCANS measured over 1 year on AS.

Events	STRATCANS Scheduled	NICE Guidelines Recommended ^1^	Difference (%)
Clinic visit	98	126	−22%
MRI	73	126	−42%
DRE	-	126	−100%

^1^ NICE: National Institute for Health and Care Excellence.

**Table 5:** Overview of the risk-based AS tools and their burden and benefit.

Model	Predicts	Performance	Burden	Benefit
PRIAS	Upgrading	AUC 0.58–0.79	+0.25 year delay ^1^	−6 biopsy sessions ^1^
Johns Hopkins	PGS	AUC 0.63–0.75	Unknown	Unknown
Canary	Upgrading	AUC 0.70	5 missed cases of upgrading ^2^	−100 follow-ups ^2^
STRATCANS	Upgrading	C-index 0.74–0.85	Unknown	−22% clinic visits and −42% MRIs ^3^

^1^ Compared to an annual schedule, measured over 10 years on AS. ^2^ Per 1000 men, measured over 4 years on AS, for the lowest risk decile. ^3^ Compared to NICE guidelines, measured over 1 year on AS.

## Data Availability

No new data were created or analyzed in this study. Data sharing is not applicable to this article.

## Abbreviations

The following abbreviations are used in this manuscript:

PCa	Prostate cancer
MRI	Magnetic resonance imaging
AS	Active surveillance
PSA	Prostate-specific antigen
DRE	Digital rectal examination
FU	Follow-up
PRISMA	Preferred Reporting Items for Systematic Reviews and Meta-Analyses
RP	Radical prostatectomy
MeSH	Medical subject headings
AUC	Area under the curve
PRIAS	Prostate Cancer Research International Active Surveillance
PASS	Prostate Active Surveillance Study
STRATCANS	Stratified Cancer Surveillance
GG	Grade group
GAP3	Third Global Action Plan
JHAS	Johns Hopkins Active Surveillance
PGS	Pathologic Gleason Score
BMI	Body mass index
UCSF	University of California, San Francisco
CI	Confidence interval
CPG	Cambridge Prognostic Groups
PSAD	Prostate-specific antigen density
C-index	Concordance index
PCSM	Prostate cancer-specific mortality
NICE	National Institute for Health and Care Excellence
PTMC	Papillary thyroid microcarcinoma
DCIS	Ductal carcinoma in situ
IBC	Invasive breast cancer
SDM	Shared decision making
SRM	Small renal masses
AI	Artificial intelligence
LB	Liquid biopsy
PRECISE	Prostate Cancer Radiological Estimation of Change in Sequential Evaluation

## Appendix A

### Literature Search

**Table A1:** Literature search.

Database Searched	Platform	Years of Coverage	Records	Records After Duplicates Removed
Medline ALL	Ovid	1946–Present	1892	1885
Embase	Embase.com	1971–Present	1902	160
Web of Science Core Collection *	Web of Knowledge	1975–Present	2565	805
Cochrane Central Register of Controlled Trials **	Wiley	1992–Present	207	125
Additional Search Engines: Google Scholar ***			185	163
Total			6751	3138

* Science Citation Index Expanded (1975–present); Social Sciences Citation Index (1975–present); Arts and Humanities Citation Index (1975–present); Conference Proceedings Citation Index—Science (1990–present); Conference Proceedings Citation Index—Social Science and Humanities (1990–present); Emerging Sources Citation Index (2005–present). ** Manually deleted abstracts from trial registries. *** Google Scholar was searched via “Publish or Perish” to download the results in EndNote. No other database limits were used than those specified in the search strategies.

**Medline**
(exp “Prostatic Neoplasms”/OR (((prostat*) ADJ3 (cancer* OR tumor* OR tumour* OR carcin* OR neoplas* OR malignan* OR adenocarin*))).ab,ti,kf.) AND (“Watchful Waiting”/OR (((activ*) ADJ3 (surveill* OR monitor*))).ab,ti,kf.) AND ((model OR models OR score* OR scoring* OR dynamic* OR stratificat* OR ((predict* OR prognost*) ADJ3 (rule*))).ab,ti,kf.) NOT (news OR congres* OR abstract* OR book* OR chapter* OR dissertation abstract*).pt.
**Embase**
(‘prostate tumor’/exp OR ‘low risk prostate cancer’/exp OR (((prostat*) NEAR/3 (cancer* OR tumor* OR tumour* OR carcin* OR neoplas* OR malignan* OR adenocarin*))):ab,ti,kw) AND (‘active surveillance’/exp OR (((activ*) NEAR/3 (surveill* OR monitor*))):ab,ti,kw) AND (‘risk model’/exp OR ‘prognostic score’/exp OR ‘risk stratification’/exp OR ‘risk stratification model’/exp OR ‘risk stratification system’/exp OR (model OR models OR score* OR scoring* OR dynamic* OR stratificat* OR ((predict* OR prognost*) NEAR/3 (rule*))):ab,ti,kw) NOT ([Conference Abstract]/lim OR [preprint]/lim)
**Web of Science**
TS=(((((prostat*) NEAR/2 (cancer* OR tumor* OR tumour* OR carcin* OR neoplas* OR malignan* OR adenocarin*)))) AND ((((activ*) NEAR/2 (surveill* OR monitor*)))) AND ((model OR models OR score* OR scoring* OR dynamic* OR stratificat* OR ((predict* OR prognost*) NEAR/2 (rule*))))) NOT DT=(Meeting Abstract OR Meeting Summary)
**Cochrane CENTRAL**
((((prostat*) NEAR/3 (cancer* OR tumor* OR tumour* OR carcin* OR neoplas* OR malignan* OR adenocarin*))):ab,ti,kw) AND ((((activ*) NEAR/3 (surveill* OR monitor*))):ab,ti,kw) AND ((model OR models OR score* OR scoring* OR dynamic* OR stratificat* OR ((predict* OR prognost*) NEAR/3 (rule*))):ab,ti,kw) NOT “conference abstract”:p)
**Google Scholar**
‘prostate|prostatic cancer|tumor|tumour|neoplasm|carcinoma|malignant|malignancy’ ‘active surveillance|monitoring’ model|models|score|scoring|dynamic|stratification ‘predict|prognostic rule’
